# Differential effects of hunger and satiety on insular cortex and hypothalamic functional connectivity

**DOI:** 10.1111/ejn.13182

**Published:** 2016-02-20

**Authors:** Hazel Wright, Xiaoyun Li, Nicholas B. Fallon, Rebecca Crookall, Timo Giesbrecht, Anna Thomas, Jason C.G. Halford, Joanne Harrold, Andrej Stancak

**Affiliations:** ^1^ Department of Psychological SciencesEleanor Rathbone BuildingBedford Street SouthLiverpoolL69 7ZAUK; ^2^ Unilever R&D3133 ACVlaardingenthe Netherlands; ^3^ Unilever R&DPort SunlightUK

**Keywords:** appetite, fMRI, homeostatic energy balance

## Abstract

The insula cortex and hypothalamus are implicated in eating behaviour, and contain receptor sites for peptides and hormones controlling energy balance. The insula encompasses multi‐functional subregions, which display differential anatomical and functional connectivities with the rest of the brain. This study aimed to analyse the effect of fasting and satiation on the functional connectivity profiles of left and right anterior, middle, and posterior insula, and left and right hypothalamus. It was hypothesized that the profiles would be altered alongside changes in homeostatic energy balance. Nineteen healthy participants underwent two 7‐min resting state functional magnetic resonance imaging scans, one when fasted and one when satiated. Functional connectivity between the left posterior insula and cerebellum/superior frontal gyrus, and between left hypothalamus and inferior frontal gyrus was stronger during fasting. Functional connectivity between the right middle insula and default mode structures (left and right posterior parietal cortex, cingulate cortex), and between right hypothalamus and superior parietal cortex was stronger during satiation. Differences in blood glucose levels between the scans accounted for several of the altered functional connectivities. The insula and hypothalamus appear to form a homeostatic energy balance network related to cognitive control of eating; prompting eating and preventing overeating when energy is depleted, and ending feeding or transferring attention away from food upon satiation. This study provides evidence of a lateralized dissociation of neural responses to energy modulations.

## Introduction

Human eating behaviour is determined and influenced by a wide array of internal and external factors. The key motivation is hunger, which (at least in lean individuals) results from a complex interplay between neurochemical compounds and signalling from homeostatically related brain structures. However, the neural basis of homeostatic networks that drive us to eat requires further elucidation in humans. This is an essential step in understanding dysfunction in appetite regulation that may lead to conditions such as obesity. Some people can resist appetitive food cues, while other people have difficulty doing so (Ouwehand & Papies, 2010; Loeber *et al*., 2013); possibly, differences in neural connectivity have a role to play (Passamonti *et al*., 2009). The insular cortex and hypothalamus are logical regions of interest, as they respond to both orexigenic and anorectic compounds (Williams *et al*., 2000; Valassi *et al*., 2008; Schloegl *et al*., 2011), and are reported as prominent activations in appetite imaging studies.

The insula is not a homogenous region of the cortex. Numerous studies have revealed functional subdivisions, with the anterior insula predominantly involved in attentional/emotional processing, and the posterior insula in sensorimotor tasks (Kurth *et al*., 2010; Cauda *et al*., 2011, 2012; Deen *et al*., 2011; Stephani *et al*., 2011; Kelly *et al*., 2012; Chang *et al*., 2013). The middle insula is less explored, but is reported to contribute to olfactory/gustatory processing (Kurth *et al*., 2010) and interoception (Kelly *et al*., 2012; Simmons *et al*., 2013). In terms of structure, cytoarchitectonic investigation of the insula cortex has revealed three insula subregions; one agranular (anterior), one dysgranular (intermediate) and one granular (posterior) in both primates (Mesulam & Mufson, 1982) and humans (Bonthius *et al*., 2005).

Regarding appetite, the anterior insula constitutes part of the primary taste cortex, and contains neurons responding to a variety of tastes and textures (Verhagen *et al*., 2004; Rolls, 2006). The mid‐insula contains neurons coding for taste intensity (Small *et al*., 2003), is activated in response to visual food stimuli (Schur *et al*., 2009; Tang *et al*., 2012) and is implicated in food craving (Pelchat *et al*., 2004). The posterior insula is activated when people deliberately induce food craving by imagining the taste and smell of food (Siep *et al*., 2012), in response to visually presented food stimuli (Britton *et al*., 2006), and to consumption of highly palatable substances (Bohon & Stice, 2011).

There is also limited evidence of functional insula lateralization in the context of appetite and food stimuli, with two recent meta‐analyses indicating greater involvement of the right insula cortex (Kurth *et al*., 2010; Tang *et al*., 2012), while another reported more involvement of the left insula (Kelly *et al*., 2012). The lack of consensus regarding insula lateralization and appetite could feasibly be due to the extensive array of experimental designs and stimuli employed between studies.

The hypothalamus plays a crucial role in food intake (Grill & Kaplan, 2002; Palkovits, 2003), and is extensively involved in homeostatic metabolic regulation (Carey *et al*., 2013; Coll & Yeo, 2013; Zhou & Rui, 2013). Eating (Thomas *et al*., 2015), and glucose (Matsuda *et al*., 1999; Smeets *et al*., 2005, 2007; Flanagan *et al*., 2012; Little *et al*., 2014) or insulin administration (Kullmann *et al*., 2013), exert significant suppressive effects on the blood oxygen level‐dependent (BOLD) signal within the hypothalamus. Additionally, the hypothalamus is physically connected to other areas involved in maintaining the homeostatic energy balance, and receives projections from the gastrointestinal tract via the brainstem (Blouet & Schwartz, 2010).

Most studies do not distinguish between the right and left hypothalamus, but some lateralized results have been reported. The left hypothalamus is sometimes found to be related to affect (Kulkarni *et al*., 2005; Cerqueira *et al*., 2008; Agroskin *et al*., 2014), though it has been shown to be smaller in patients with anorexia (Titova *et al*., 2013), and modulated by leptin (Rosenbaum *et al*., 2008). The right hypothalamus seems more related to homeostatic appetitive processing, demonstrating activation to visual food stimuli (Rosenbaum *et al*., 2008), especially those depicting food with a high energy content (van der Laan *et al*., 2011), or following weight loss in obesity (Hinkle *et al*., 2013). It is at least partially responsible for the anorectic response to acute nicotine administration (Kroemer *et al*., 2013), and its functional connectivity is modulated by leptin (Hinkle *et al*., 2013).

The current study was designed to investigate appetite‐induced functional connectivity changes in anterior, middle and posterior seeds in the left and right insula, and in the left and right hypothalamus seeds, using resting state functional magnetic resonance imaging (fMRI). Resting state refers to a paradigm whereby participants lie quietly in the scanner without performing any tasks. Functional connectivity measures track temporal correlations in BOLD fluctuations between brain areas, allowing for the identification of coherent brain area networks. It was hypothesized that the seeds will exhibit differential patterns of functional connectivity depending on participants’ satiation.

## Materials and methods

### Participants

MRI safety screening was carried out by a radiographer, and an additional thorough medical screening was completed by the experimenter. The Three Factor Eating Questionnaire – Revised (TFEQR18: Karlsson *et al*., 2000) was completed at the screening. Nineteen healthy Caucasian volunteers (nine male) with a normal body mass index (BMI; World Health Organisation, 2006) took part in this study. The average age of the participants was 24.8 ± 3.8 years (mean ± SD). Participants gave their written informed consent and the study was conducted in accordance with the Declaration of Helsinki. Local ethical approval was obtained from the University of Liverpool Research Ethics Committee.

### Procedure

Participants attended two sessions, separated by 9.2 ± 4 days. On the day before both sessions, participants were reminded not to exercise more than they would normally, and not to eat or drink anything but water after midnight. Compliance was assessed using diary entries and blood glucose testing upon arrival at the imaging facility at 09:30 h or 10:00 h.

Session order was counterbalanced across participants. For the fasted session, they completed the MRI scans after a minimum of a 9.5 h overnight fast. For the fed session they were given a fixed load breakfast after their overnight fast, and then completed the MRI scans after a short (approximately 20 min) delay. The total energy content of the fixed load breakfast was 531 kcal (26.55% of the recommended daily intake) for females and 670 kcal (26.8% of the recommended daily intake) for males, and consisted of cornflakes, semi‐skimmed milk, toast, margarine, strawberry jam and orange juice.

Measures of hunger, desire to eat and prospective consumption (how much food could potentially be eaten) were taken using 100 mm visual analogue scales (VAS; Stubbs *et al*., 2000) immediately prior to the MRI scans in both sessions. Blood glucose samples were obtained using a handheld blood glucose monitor (Model: Accu‐Chek Aviva, Roche Diagnostics, West Sussex, UK) immediately prior to the completion of the VAS scales. The Profile of Mood States (McNair *et al*., 1981) was employed to measure participants’ mood before the scans in both sessions.

### Image acquisition

MRI scans were undertaken using a whole‐body Siemens Trio 3T scanner (Siemens, Erlangen, Germany) with an eight‐channel radiofrequency head‐coil. Foam padding was used to restrict head movement. A T2‐weighted sequence was used to acquire functional resting state images covering the whole brain (32 axial slices), TR = 2000 ms, TE = 30 ms, slice order = interleaved ascending, flip angle = 90°, field of view = 192 mm, slice thickness = 3.5 mm (0.7 cm gap), voxel size at acquisition = 3.0 × 3.0 × 3.5 mm.

In the scanner, participants were asked to relax, close their eyes and refrain from falling asleep. After the scan they were asked to recall what they were thinking about during the scan, and give a rating between 1 (‘not at all’) and 7 (‘constantly’) in response to the question ‘How much were you thinking about eating or food?’.

### Data analysis

SPM8 (UCL, UK: www.fil.ion.ucl.ac.uk/spm) running on Matlab version R2012a (MathWorks, Natick, MA, USA) was used to preprocess the data. Images were first slice‐timing corrected, then realigned to the first slice and unwarped, normalized to the template EPI image, and smoothed using an 8‐mm full‐width half‐maximum Gaussian kernel.

Preprocessed images were imported to the functional connectivity toolbox ‘Conn’ v.13 ( www.nitrc.org/projects/conn; Whitfield‐Gabrieli & Nieto‐Castanon, 2012). The grey matter, white matter and cerebrospinal fluid masks were produced by segmenting SPM8's template EPI image using the segmentation routine in SPM8, and resliced to match the image dimensions of the preprocessed functional images. No ‘partition clean‐up’ was performed, and the ICBM European brains template was used for affine regularization.

Insula seed masks were produced using MarsBaR ( http://marsbar.sourceforge.net/; Brett *et al*., 2002). A study on functional differentiation within human insula (Cauda *et al*., 2011) was used to define left and right anterior, middle (called ‘transitional zone’ in the original paper), and posterior insula seeds for the current analysis. Each insula seed was comprised of multiple 5‐mm^3^ clusters, detailed in Table 1.

**Table 1:** Coordinates of the individual 5‐mm^3^ clusters defined by Cauda *et al*. (2011), grouped into the left or right anterior insula seed, middle insula seed or posterior insula seed

Seed	Clusters	Left X	Y	Z	Cluster (k)	Right X	Y	Z	Cluster (k)
Ant. Ins.	1	−34.5	12.5	−2.5	–	34.5	12.5	−2.5	–
	2	−36.5	4.5	−3	–	38.5	5.5	−2.5	–
	3	−30.5	18.5	5.5	–	34.5	16.5	5.5	–
	4	−32.5	9	5	–	36.5	7	5	–
	5	−30.5	9	11.5	936	32.5	9	11.5	864
Mid. Ins.	1	−36.5	−0.05	4.5	–	38.5	−0.5	4.5	–
	2	−34.5	−3	11	432	34.5	−3	11	432
Post. Ins.	1	−36.5	−7.5	−3.5	–	36.5	−4.5	−3	–
	2	−36.5	−10	4	–	38.5	−8	4	–
	3	−34.5	−13	10	648	34.5	−11	10.5	648

**Figure 1:**
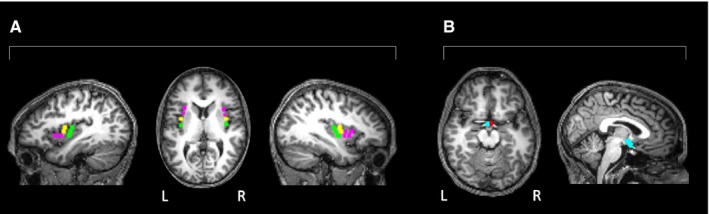
Seeds superimposed on a typical participant's T1 scan. (A) anterior insula (magenta), middle insula (yellow), and posterior insula (green); (B) left hypothalamus (blue), right hypothalamus (red). L, left; R, right.

Coordinates were given in Talairach space in the original paper; those presented here have been transformed into MNI space using the Matlab script ‘tal2mni’ (Brett, 2001), and rounded up or down to the nearest 0.5 mm. Cluster (*k*) refers to the number of voxels overall within the left or right anterior, middle, and posterior insula seeds. The clusters are shown superimposed on a randomly selected participant's structural scan in Fig. 1.

Ant. Ins., right anterior insula seed; Mid. Ins., middle insula seed; Post. Ins., posterior insula seed.

Individual participants’ realignment parameter files were added as first‐level covariates. Data were initially bandpass filtered from 0.008 to 0.09 Hz in order to remove noise and low‐frequency drift. Finally, the signal from white matter and cerebrospinal fluid was entered as confounds and removed using linear regression.

The remaining data were entered into first‐level analysis in a paired *t*‐test design. Individual seed‐to‐voxel connectivity maps for each insula seed and each participant were generated separately for the fasted and fed sessions. For second‐level analysis, one‐sided *t*‐tests were employed to examine changes in seed‐to‐voxel connectivity between sessions. Glucose scores were entered as a second‐level covariate, expressed as a function of Δ (difference) between the sessions. The results were thresholded at *P* < 0.05 FWE corrected in order to account for multiple comparisons. While the FWE correction for the height threshold is generally considered sufficient to mitigate the risk of a type I error, an additional FWE corrected extent threshold of 100 voxels was applied as an extra precaution. The FWE correction was applied separately for each seed region of interest across its multiple target voxels.

The analysis was repeated with the hypothalamus seeds. It was identical to the insula connectivity analysis in all aspects, except for the seeds themselves. The seed masks consisted of tracings around the bilateral hypothalamus (as defined by the AAL atlas; Tzourio‐Mazoyer *et al*., 2002) produced by Sudheimer *et al*. (2015). The results were again thresholded as described above.

**Figure 2:**
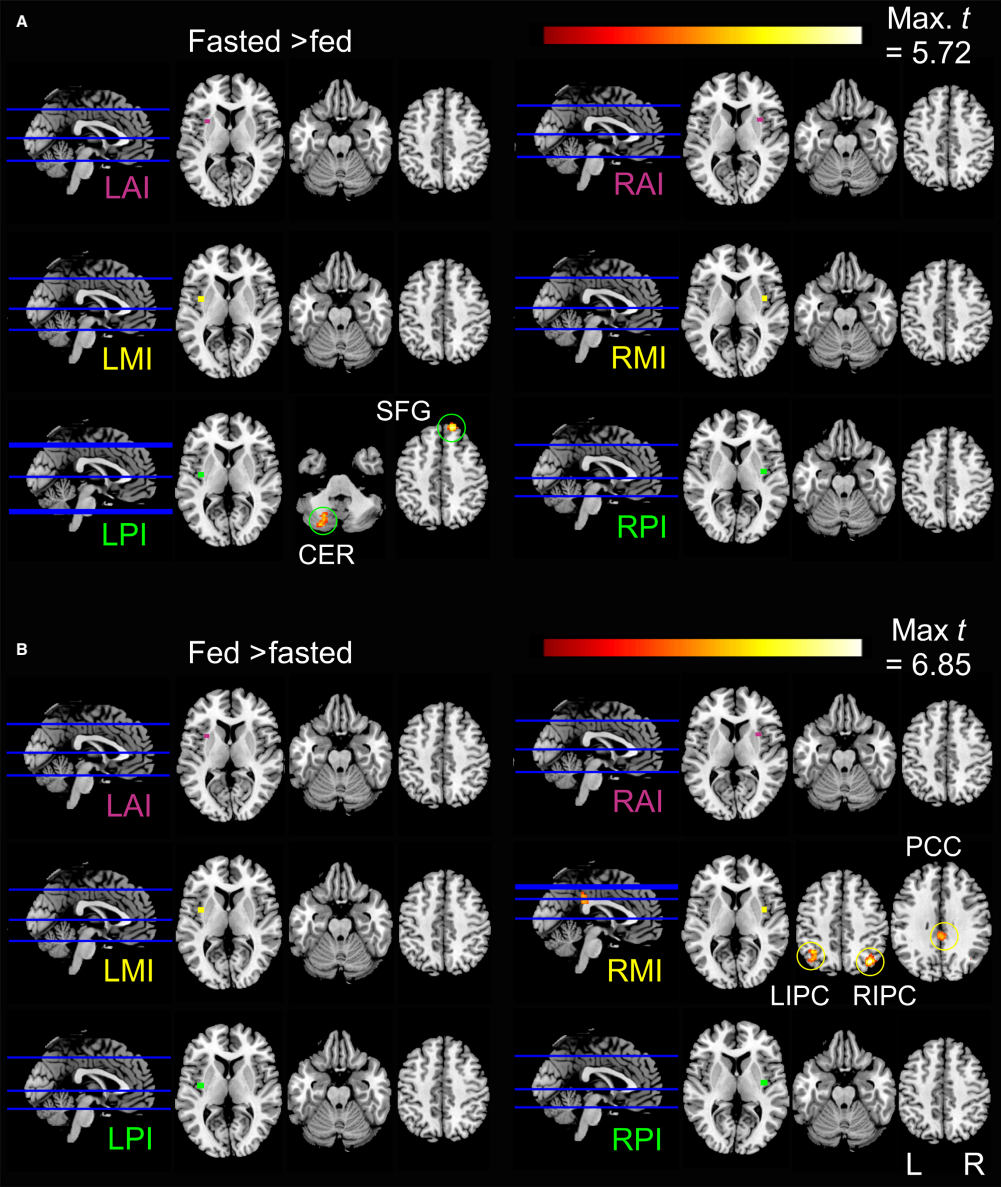
Slice locations, insula seeds and connectivity maps. L, left; R, right; AI, anterior insula; MI, mid insula; PI, posterior insula; CER, cerebellum; SFG, superior frontal gyrus; IPC, inferior parietal cortex; PCC, posterior cingulate cortex.

Figures 1 and 2 were produced using xjView v.8.12 ( www.alivelearn.net/xjview8/) and MRIcron ( www.nitrc.org/projects/mricron; Rorden *et al*., 2007).

## Results

MNI coordinates of seeds and their functionally connected areas are shown in Table 2.

**Table 2:** MNI coordinates refer to the peak activated voxels

Seed	Cluster	Cluster MNI *x*,* y*,* z* (mm)	*k*	*t*_1,18_	P‐FWE cluster
Fasted > Fed					
LPI	SFG	16, 50, 50	142	5.72	0.03
	Cerebellum	−26, −80, −38	208	4.72	0.004
LHYP	IFG	36, 32, −14	127	5.56	0.03
Fed > Fasted					
RMI	Cingulate	2, −38, 38	206	5.02	0.004
	L IPC	−40, −54, 48	236	5.05	0.002
	R IPC	38, −68, 42	302	6.85	0.0003
RHYP	L SPC	−6, −58, 68	162	2.33	0.02

FWE, family‐wise error; IFG, inferior frontal gyrus; *k*, cluster size (voxels); LHYP, left hypothalamus; L IPC, left inferior parietal cortex; LPI, left posterior insula; L SPC, left superior parietal cortex; MNI, Montreal Neurological Institute; RHYP, right hypothalamus; R IPC, right inferior parietal cortex; RMI, right middle insula; SFG, superior frontal gyrus.

### Self‐report measures and glycaemia

In comparison to the fed session, during the fasted session participants reported feeling significantly more hungry (mean increase of 53.46 ± 18.5 on the VAS scales; *t*_18_ = 12.53, *P* < 0.001) before the scan, and more distracted by thoughts of food or eating (*t*_18_ = 4.31, *P* < 0.001) during the scan. Participants also recorded significantly lower blood glucose levels before the fasted scan (mean fasted reading = 5.07 ± 0.35 mm; mean fed reading = 6.79 ± 0.72 mm;* t*_18_ = −10.58, *P* < 0.001). Profile of Mood States scores were not significantly different between sessions (*t*_18_ = −1.54, *P* > 0.05).

### Functional connectivity of insula

When participants were fasted, enhanced functional connectivity between the left posterior insula and right superior frontal gyrus (SFG), and left posterior insula and left cerebellum were observed (Fig. 2A). When participants were fed, they exhibited enhanced functional connectivity between the right mid‐insula and left inferior parietal cortex (IPC), right IPC and cingulate cortex (Fig. 2B). The enhanced functional connectivities between the left posterior insula and SFG, and right middle insula and cingulate cortex disappeared when glycaemia was added as a second‐level covariate. This finding indicates that the augmented connectivities were at least partially related to differences in blood glucose levels across sessions.

To further explore the connectivities dependent on blood glucose, correlations between Δ functional connectivities of the left posterior insula–SFG and Δ glycaemia (Fig. 4A), and between the right middle insula–cingulate cortex and Δ glycaemia (Fig. 4B) were plotted.

### Functional connectivity of the hypothalamus

**Figure 3:**
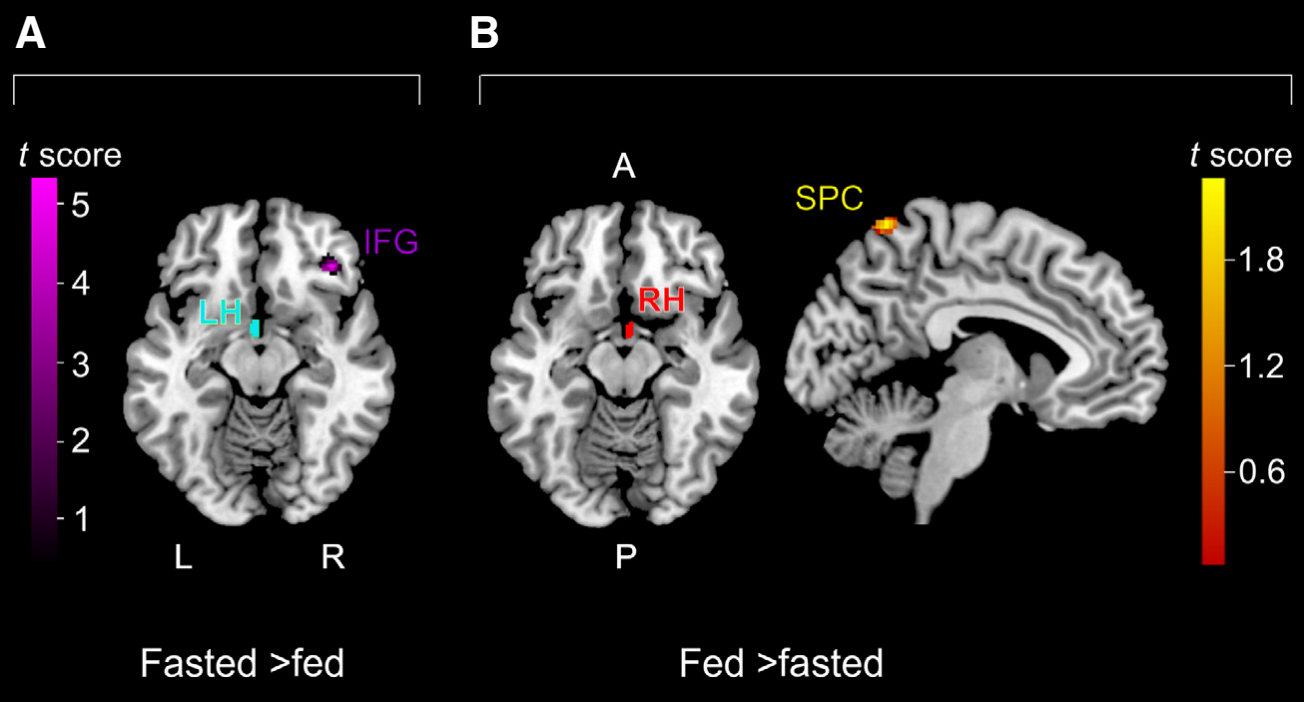
Hypothalamus seeds and connectivity maps. L, left; R, right; A, anterior; P, posterior; IFG, inferior frontal gyrus; SPC, superior parietal cortex.

During fasting, there was enhanced functional connectivity between the left hypothalamus and right inferior frontal gyrus (IFG; Fig. 3A); and, during satiety, between the right hypothalamus and a cluster in the left superior parietal cortex (SPC), spanning the left postcentral gyrus and precuneus (Fig. 3B). Δ Glycaemia was at least partly responsible for the enhanced connectivity between the left hypothalamus and IFG, and the right hypothalamus and SPC.

**Figure 4:**
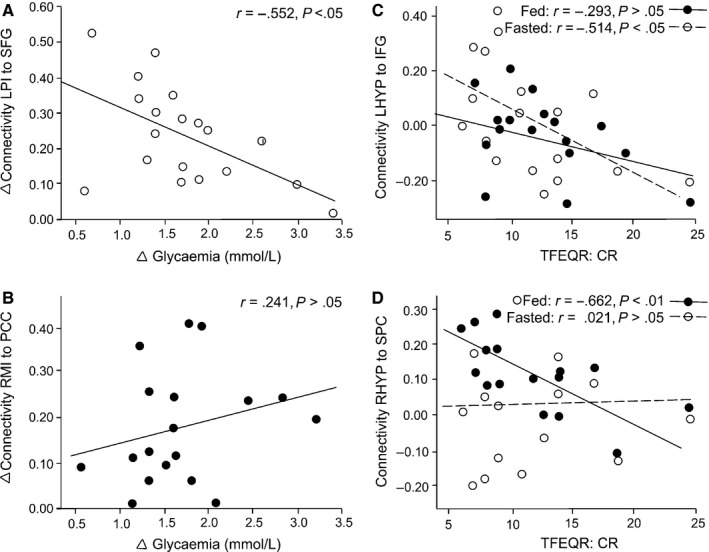
(A) *y* axis is Δ connectivity between sessions from left posterior insula (LPI) to SFG; (B) *y* axis is Δ connectivity between sessions from right middle insula (RMI) to posterior cingulate cortex (PCC); (C) is the connectivity between left hypothalamus/IFG and the cognitive restraint subscale of the TFEQR18; (D) is the connectivity between right hypothalamus/SPC and the cognitive restraint subscale of the TFEQR18.

During the fasted session, the correlation between the left hypothalamus/IFG functional connectivity and the Cognitive Restraint scale of the TFEQR18 was significant (*r* = −0.514, *P* < 0.05), while during the fed session the correlation between the right hypothalamus/SPC functional connectivity and the Cognitive Restraint scale of the TFEQR18 was also significant (*r* = −0.662, *P* < 0.01). These correlations are presented in Fig. 4C and D, respectively.

## Discussion

In support of the current hypothesis, the resting state functional connectivities of some insula regions and the right and left hypothalamus were altered by changes in the homeostatic energy balance.

### Insula

During the fasted session, the left posterior insula displayed enhanced connectivity with the cerebellum and right SFG. The posterior insula has been shown to be activated during hunger (Tataranni *et al*., 1999), deliberately induced food craving (Siep *et al*., 2012), in response to a preferred food odour (Bragulat *et al*., 2010) and on receiving an appetitive drink (Bohon & Stice, 2011), and represents a wide range of homeostatically related sensations in addition to hunger, such as thirst, dyspnoea (hunger for air) and pain (Craig, 2002, 2003).

The cerebellum contains a dense population of leptin receptors (Couce *et al*., 1997; Burguera *et al*., 2000; Harvey, 2003, 2007; London *et al*., 2011), is activated during hunger (Tataranni *et al*., 1999) and exhibits significant decreases in activation after feeding (Gautier *et al*., 2000, 2001; Del Parigi *et al*., 2002; Haase *et al*., 2011). The enhanced functional connectivity seen between the left posterior insula and cerebellum suggests a homeostatic circuit motivating food ingestion after fasting.

The SFG is part of a frontal network that represents the motivational or reward value of different foods (Killgore *et al*., 2003; Hare *et al*., 2009). It has been frequently theorized to be involved in inhibiting approaches to food (Tataranni *et al*., 1999; Gautier *et al*., 2000; DelParigi *et al*., 2006; McCaffery *et al*., 2009; Batterink *et al*., 2010), and is also activated in response to appetitive stimuli when participants are fasted (Burger & Stice, 2011; Malik *et al*., 2011; Martens *et al*., 2013). It therefore appears to serve a dual purpose, motivating either approach to food or restraint from eating, according to the homeostatic energy balance. While no appetitive stimuli were presented during the resting state scan, participants nevertheless reported experiencing significantly more thoughts about food or eating during the fasted session scan, raising the possibility that their thoughts acted as appetitive stimuli.

The enhanced connectivity between the posterior insula and SFG during the fasted session was abolished when Δ glycaemia was added as a covariate, indicating that changes in blood glucose levels were at least partially responsible for the augmented functional connectivity. Taken together, the results suggest that fasting‐induced alterations in functional connectivity appear to be related to alleviating an acute homeostatic energy deficit.

During the fed session, the right mid‐insula was more strongly functionally connected to the left and right IPC, and to the cingulate cortex. Many studies have cited the left IPC, right IPC and cingulate cortex as being part of the default mode network (DMN; Greicius *et al*., 2003; McKiernan *et al*., 2003; Fox *et al*., 2005; Fransson, 2005; Vincent *et al*., 2006; Laird *et al*., 2009; McFadden *et al*., 2013). The DMN activations represent the ‘resting state’ of the brain, deactivating in response to task demands. It appears to underlie self‐referential and memory‐related processes, with memory consolidation (Miall & Robertson, 2006), remembering and thinking about the future (Buckner & Carroll, 2007), and mind‐wandering (Mason *et al*., 2007) being among the observed functions of the DMN.

Other functional connectivity studies report resting state connectivity between the insula and areas of DMN (Taylor *et al*., 2009; Zou *et al*., 2009; Li *et al*., 2012b; Liang *et al*., 2013). While research has demonstrated the involvement of the mid‐insula in feeding behaviour (Small *et al*., 2001; Li *et al*., 2012a), and some association between DMN activity and obesity (Tregellas *et al*., 2011; Kullmann *et al*., 2012; McFadden *et al*., 2013), it is likely in the context of the current study that its enhanced functional connectivity with DMN areas has more to do with introspective processes, as the homeostatic energy balance had already been restored. The mid‐insula has previously been cited as a region strongly associated with interoception (Kelly *et al*., 2012; Simmons *et al*., 2013), and in the current study with healthy participants, the enhanced mid‐insula to default mode structures connectivity was accompanied by a reduction in self‐reported thinking about food and eating.

### Hypothalamus

The left hypothalamus was more strongly functionally connected to the IFG during the fasted session. Previous studies suggest that the activation of the IFG is related to cognitive control (Hare *et al*., 2009; Sundermann & Pfleiderer, 2012). It is involved in suppressing the desire for food (Hollmann *et al*., 2012) and successfully resisting temptation (Lopez *et al*., 2014), and is more strongly activated by food stimuli in successful weight loss maintainers than in obese or normal weight participants (Sweet *et al*., 2012). Additionally, IFG grey matter volume (Brooks *et al*., 2013) and activation to satiety (Le *et al*., 2009) is significantly reduced in obesity. There is also a relationship between hypoactivation of prefrontal cortices and elevated BMI (Le *et al*., 2006, 2007; Volkow *et al*., 2009; Page *et al*., 2011; Willeumier *et al*., 2011). In the current study, IFG was more strongly functionally connected to the left hypothalamus when participants were fasted, and this enhanced functional connectivity was significantly correlated with Cognitive Restraint subscale scores on the TFEQR18. Rather than driving food consumption when there is an energy deficit, IFG appears to attempt to ensure that overfeeding does not occur. It seems likely that the hypothalamic drive to eat is being tempered (Tataranni *et al*., 1999), though the analysis method used here does not allow for the specification of directional modulation.

During the fed session, the right hypothalamus was more strongly functionally connected to a cluster in the SPC, which encompassed the postcentral gyrus and precuneus. The precuneus is deactivated in response to satiation (Gautier *et al*., 2000), and consciously suppressing food craving also reduces activation in the precuneus (Yokum & Stice, 2013). Both areas are altered in obesity; the left postcentral gyrus grey matter volume is reduced (Brooks *et al*., 2013) and the precuneus shows a reduced response to visual food stimuli (Heni *et al*., 2014).

Taking this and previous findings into account, the enhanced functional connectivity that was observed appears to have been modulated by the homeostatic energy balance, especially because the addition of the glycaemia covariate cancels this enhanced connectivity. In the absence of an energy deficit, it is possible that the functional connectivity between the right hypothalamus and SPC might represent the suppression of the eating drive. The strong correlation between this functional connectivity during the fed session and the Cognitive Restraint subscale of the TFEQR18 lends support to this hypothesis. It might also be the case that when the energy balance was restored, the participants were able to move their attention away from food. Participants reported being significantly less distracted by thoughts of food or eating during the fed session, and the SPC is considered to be a core area involved in many types of attentional processing (Corbetta *et al*., 2000).

### Limitations

Blood glucose samples were obtained using a handheld blood glucose monitor and, as such, serum samples are not available for further analysis. Blood glucose sampling was included initially as a crude verification of clear differences between the fed and fasted states. Ultimately it proved a much more important measure than originally anticipated, and taking a comprehensive profile of blood serum may well have provided additional interesting results. However, some inferences regarding commonly studied appetite‐related hormones can be drawn on the basis of other research using similar designs and healthy weight participants.

Alterations in free leptin levels are associated with activation changes in the insular cortex and other homeostasis‐related brain areas (Farr *et al*., 2014; Olivia *et al*., 2014). However, leptin has previously been shown to remain at a stable level for at least 2 h after a test meal (Korbonits *et al*., 1997; Carlson *et al*., 2009), and only begins to fall significantly after about 16 h of fasting (Korbonits *et al*., 1997). Both these timing parameters exceed those utilized in the present study (minimum 8 h fast; scanned approximately 20 min after breakfast), and therefore it seems unlikely that changes in leptin were a factor in the current results.

Feeding has a more acute effect on ghrelin release; the levels appear to be significantly decreased approximately 20–30 min after a test meal (Carroll *et al*., 2007; Carlson *et al*., 2009). Insulin concentration is significantly increased from baseline fasting levels approximately 15 min after a test meal, and does not begin to drop noticeably for at least 30 min (Carroll *et al*., 2007; Carlson *et al*., 2009). Both compounds are associated with the modulation of the insula cortex and hypothalamic activation (Nakazato *et al*., 2001; Malik *et al*., 2008; Wang *et al*., 2008; Berthoud, 2011; Schloegl *et al*., 2011; Li *et al*., 2012a; Kullmann *et al*., 2013), and both exhibit peak changes in concentration at about the length of time after a meal that the current participants were being scanned. It is therefore likely that there are unaccounted for additional hormonal factors influencing or being influenced by changes in these functional connectivities.

## Conclusion

The insula and hypothalamic functional connectivity patterns are altered by changes in the homeostatic energy balance. Functional connectivity with subregions of the frontal cortex seems to form part of a circuit motivating feeding, preventing overeating and terminating feeding upon satiation. Further research could examine functional connectivity differences between lean and obese participants; possibly alterations in insula or hypothalamus functional connectivity would be found in obesity. If so, this could represent a new target for interventions.
